# Sedimentation Stability of Magnetorheological Fluids: The State of the Art and Challenging Issues

**DOI:** 10.3390/mi13111904

**Published:** 2022-11-03

**Authors:** Seung-Bok Choi

**Affiliations:** 1 Department of Mechanical Engineering, The State University of New York, Korea (SUNY Korea), 119 Songdo Moonhwa-ro, Yeonsu-gu, Incheon 21985, Korea; seungbok.choi@sunykorea.ac.kr; 2 Department of Mechanical Engineering, Industrial University of Ho Chi Minh City (IUH), 12 Nguyen Van Bao Street, Go Vap District, Ho Chi Minh City 70000, Vietnam

**Keywords:** magnetorheological (MR) fluid, sedimentation stability, magnetic particle, carrier liquid, additive, particle treatment, additive recipe

## Abstract

Among the many factors causing particle sedimentation, three principal ingredients are heavily involved: magnetic particles, a carrier liquid (base oil), and additives (surfactant). Therefore, many works have been carried out to improve the sedimentation stability of magnetorheological fluids (MRFs) by adopting the three methods. In the particle modification stage, the weight concentration, size distribution, particle shape, coated materials, and combinations of different sizes of the particles have been proposed, while for the modification of the carrier liquid, several works on the density increment, wettability control, and the use of natural oils, lubricant oil, grease, and ethyl- and butyl-acetate oils have been undertaken. Recently, in certain recipes to improve sedimentation stability, some additives such as aluminum stearate were used to increase the redispersibility of the aggregated particles. In addition, several works using more than two recipes modifying both the particles and base oils are being actively carried out to achieve higher sedimentation stability. This review article comprehensively introduces and discuses the recipes to improve sedimentation stability from the aspects of the three ingredients. A few conceptual methodologies to prevent the sedimentation occurring via a bottle’s storage on the shelves of the application systems are also presented, since, to the author’s knowledge, there has not been a report on this issue. These are challenging works to be explored and developed for successful application systems’ MRFs.

## 1. Introduction

Currently, many different smart materials are being developed and studied since their inherent properties are very effective for making several different actuators or sensors applicable to dynamic systems associated with passive and active controllers to achieve high performance. Among the many smart materials, magnetorheological fluids (MRFs) containing three principal ingredients—magnetic particles, carrier fluids, and additives—are actively studied to improve the field-dependent magnetorheological (MR) effect [1,2]. MRFs can have several salient properties that are beneficial for creating various control systems in passive or semi-active manners. The saliant properties of MRF include a fast response time to the applied input, a wide frequency spectrum due to the fast response time, a wide domain of controllable force (or torque, moment, etc.), and fail-safe design functions due to the original viscosity of the carrier liquid. Therefore, numerous application systems have been developed so far, and some systems such as MR shock absorbers for vehicle suspension systems have already been commercialized. Many of the application systems utilizing MRFs are closely related to vibration control, but some of these applications include soft robots, medical devices, and rehabilitation mechanisms. Specifically, the MRF applications for vibration control include dampers, mounts and brakes [3,4,5,6,7,8], and specific medical and rehabilitation devices, which are introduced in [9,10,11,12,13,14]. It is noted here that there are many review articles focusing on MRF applications including damper systems, medical devices, and seismic mitigation [15,16,17,18,19]. Despite the many studies on the prototypes of application systems in laboratories, specific research on their practical realization is considerably rare. This directly indicates that the MRFs developed so far do not sufficiently meet certain conditions required for practical systems subjected to hard operating conditions and uncertainties including external disturbances. In fact, there are several challenging issues to be resolved for the successful commercialization of numerous application systems using MRFs. Some critical areas include particle sedimentation, durability, sealing to avoid the liquid leakage, integration with microcontrollers, operability under wider temperature ranges, etc. Among these, particle sedimentation due to the density mismatch between the particle and carrier liquid is the most serious problem to be resolved for commercial applications of MRF-based systems in practical environments.

Consequently, the main technical contribution of this work is to comprehensively investigate and summarize the recent studies on the improvement of the sedimentation stability in MRFs. As mentioned, one method to reduce sedimentation is to modify the particles. For example, the addition of nanosized particles, the use of new processing techniques to make carbonyl iron (CI) particles with amino groups, mixing micro-sized plate-like particles, the addition of spherical fillers with nanosized fumed silica, the use of Fe_3_O_4_ nanospheres, and the use of iron-doped poly-pyrrole nanoparticles have been developed and hence discussed in this review article addressing the techniques for modifying the particles in detail. As for the carrier liquid, in order to enhance its sedimentation stability, density modification, the change of the kinematic viscosity, the use of a green carrier liquid, the use of grease, the combination of silicone oil and a lubricant named molybdenum disulfide, and the use of boron nitride have been studied, and hence the specific recipes will be discussed. On the other hand, many studies using certain additives have been undertaken to improve sedimentation stability maintaining the field-dependent rheological properties such as the yield shear stress. Some of additives frequently used include aluminum stearate, the acrylic acid polymer, a silane-coupling agent, dimer acid, the styrene fumarate copolymer, the use of clay-based additives, dodecyl benzoate, polyethylene glycol, and sodium dodecyl sulfate. There are also studies on the sedimentation effects of coated particles using multi-wall carbon nanotubes (MWCNT). Recently, several recipes combining more than two recipes have been studied to reduce the particle sedimentation. For example, the combination of carbonyl iron particles (CIP), silicone oil, coating with guar gum, stearic acid additive, and submicron organoclays additives, as well as the mixing of lithium zinc ferrite, nickel zinc ferrite, cottonseed oil, carbon globules synthesized from toluene, and coatings with stearyl acyl ethylenediamine triacetate, have been studied and used; thus, they will be discussed in detail in this review article. 

**Figure 1:**
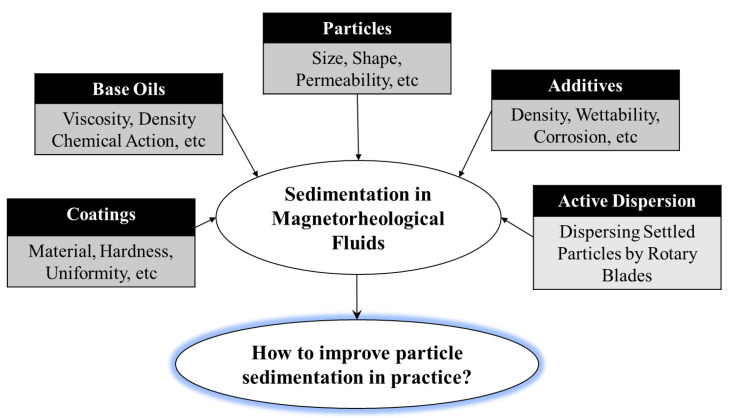
Various recipes to improve the sedimentation stability of MRFs.

Figure 1 presents several recipes detailing methods to improve the sedimentation stability of MRFs. Most of the recipes proposed so far are passive, in which there is no action to reduce the particle sedimentation during the operation of the application systems or devices. One active method to reduce particle sedimentation is to use an active dispersing mechanism, where a small-sized rotary blade is located inside of the piston. In this mechanism, the settled particles are actively dispersed by the rotating speed and angle of the blades during operation. However, so far, there is no solution that prevents the sedimentation problem regarding storage (wherein bottles filled with MRF are stored on shelves) for the practical use of application systems filled with MRF. These challenging issues are to be conceptually discussed in this article. In fact, a couple of concepts to reduce particle sedimentation from bottle storage for use in real application systems are suggested and discussed. Notably, this review article investigates and discusses the MRFs’ sedimentation problem focusing on both each principal ingredient of MRFs and a practical point of view for the successful marketing of products with diverse application systems. Therefore, the author believes that this review article is timely, good, and helpful for many potential scholars who are working on the development of advanced MRFs as well as high-performance application systems. 

## 2. Improvement of Sedimentation Stability

### 2.1. Particle Modification

**Figure 2:**
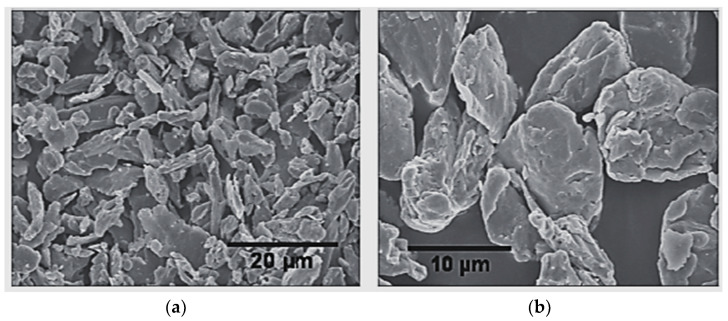
Morphology of the microstructure of iron plate-like particles in (**a**) small-sized particles and (**b**) large-sized particles [24].

As mentioned in the Introduction, there are several ways to reduce sedimentation by modifying particles in terms of their size, shape, permeability, and so forth. In this review, some of the methods popularly studied by many researchers are investigated and discussed in a chronological manner. One of the easiest methods is the addition of Co-gama-Fe_2_O_3_ and CrO_2_ particles to CIP [20]. The addition of such needle-like particles improves stability against rapid sedimentation because the added particles play a role in the steric repulsion between the relatively large CIPs maintaining a high yield stress at a high magnetic field strength. S. T. Lim et al. [21] mixed submicron-sized particles of fumed silica with CIP for the enhancement of sedimentation stability. The addition of the spherical particles not only reduced the degree of sedimentation, especially in the beginning, but also improved the flocculation stability of the original CIP-based MRF without any noticeable change in the field-dependent MR effect. The use of the CI micron-particles functionalized with reactive silane, which are coated with cholesteryl groups, can increase sedimentation stability [22]. In this case, the sedimentation ratio of the pure CI particles is very fast, while the modified particles exhibit slower sedimentation at the very beginning. K. Shah et al. [23] investigated the sedimentation effect on the field-dependent-damping force of an MR damper filled with plate-like particles. The shape of the particles play an important role in improving stability against rapid sedimentation. The degree of sedimentation stability is increased, maintaining the field-dependent-damping-force and response time of the MR damper. Particle size can affect sedimentation stability and the field-dependent rheological properties; hence, it has been studied. Figure 2 presents the size and surface morphology of the iron plate-like particles [24]. The average size of a small particle is 2 μm, while that of a large particle is 19 μm. In this study, the settling rate of the small particles was identified as 1.33%/day, while 1.38%/day was determined for the large particles, as expected. Jonkkari et al. [25] studied this effect using three different concentrations of particles: 15% (weight) microparticles, 15% microparticles with 5% nanoparticles, and 15% microparticles with 10% nanoparticles. The prepared particles were mixed with two carrier liquids: silicone oil and ionic oil. The ionic carrier liquid and addition of 10% nanoparticles mostly reduces the sedimentation rate, while the field-dependent dynamic yield stress is slightly less than that of the original MRF consisting of 15% microparticles only. G. Wang et al. [26] proposed magnesium ferrite (MgFe_2_O_4_) nanocrystal clusters synthesized with an ascorbic acid-assisted solvothermal method to enhance sedimentation stability. After the observation of the microstructures and magnetic properties of the MgFe_2_O_4_ nanocrystal clusters, it was mixed with silicone oil to prepare MR fluid. The proposed fluid exhibits better sedimentation stability than conventional CIP–silicone oil fluid due to the reduced density mismatch between the particles and the carrier medium. However, the field-dependent yield stress of the proposed fluid is lower than the conventional one. In order to investigate sedimentation under gravity, T. Kikuchi et al. [27] tested four MRF samples that were different in terms of their particle concentrations and carrier viscosity. The test was carried out under high centrifugal acceleration and the results were compared with the degree of sedimentation under natural gravity. To guarantee a high aspect ratio and evaluate the state of sedimentation in the small gap, a capillary with a small diameter (0.2 mm) was used. As expected, the higher the particle fraction yield, the lower the sedimentation stability. It is interesting to note that the normalized yield stress of the four samples have nearly the same field-dependent yield stress under high acceleration. 

On the other hand, a study on the effect of thermal conductivity on sedimentation has also been investigated in [28]. To achieve this target, a series of MRF samples were made using normal CIP and hydraulic oil, and then by adding three different nano-sized particles: copper (CU), aluminum (AL), and fumed silica (SiO_2_). Two significant results were found from this work: the thermal conductivity of the proposed samples is much better than MRF 132DG, showing a 148% increment when the volume of the magnetic particles is 40%. The sedimentation rate of the proposed samples is reduced by 9% with a 40% volume of magnetic particles. The field-dependent shear stress is not deteriorated compared with the commercial MRF-130DG. J. Vepys et al. [29] investigated the effect of the particle sedimentation of MRF on the torque moment applied to brakes. The torque moment was tested as a function of the shear rate. Five different magnetorheological fluids—MRF-140CG and MRF-122EG from Lord Corporation, USA; MRHCCS4-A and MRHCCS4-B from Liquids Research Company, UK; and MUDZH-3, made in Luikov Heat and Mass Transfer Institute, Belarus—were tested. The sedimentation of MRF was tested using electric resistivity by measuring the different signals obtained from the top and bottom sensors. It was found that sedimentation influences the torque moment of the brake, for which the MUDZH-3 fluid exhibits the best sedimentation stability. Sedimentation models have been also studied by several researchers. In order to ascertain a model’s accuracy, Zhang et al. [30] prepared and tested four MRF samples, which had different particle volume fractions and three different surfactant mass fractions. The samples were prepared, and the morphologies of the bare CI and surface-modified CI particles were observed. It was identified that the Dick model is more suited to the measurement of the sedimentation of the proposed fluid rather than the Vesilind, Richardson, and Zaki models since this model can accurately establish the propagation velocity formula of the volumetric concentration of the particles. An investigation of the effect of mixing suspended nanoparticles into a bi-disperse MR fluid on sedimentation was also studied [31]. The bi-disperse fluid was modified using two different shapes of magnetic nanoparticles: spheres and hexagonal platelets. In addition, as a reference, a fluid containing micro-sized CIP was made. It was found that the presence of the spherical nanoparticles exhibits the slowest sedimentation, while the fluid based on the soft CIP with the nanospheres is more stable than the hard CIP-based fluid since the sedimentation rate of the soft CIP-based fluid is slow over a long period. J. Choi et al. [32] investigated the MR properties of a carbon nanotube (CNT)-Co0.4Fe0.4Ni0.2 composite suspension with respect to enhancing both the MR’s effect and sedimentation stability. Unexpectedly, a very high field-dependent yield stress, which was 13 times higher than CNT-Fe_3_O_4_ suspension, was identified. In addition, the low density of the CNT-Co0.4Fe0.4Ni0.2 nanocomposite suspension shows much better stability than the fluid containing only Co0.4Fe0.4Ni0.2. This is because that the synergistic effect between the high aspect ratio of the CNTs and the strong magnetic polarization of the Co0.4Fe0.4Ni0.2 lead to the stronger MR performance of the nanocomposite particle suspension. In order to enhance sedimentation stability, Fe_3_O_4_ nanospheres were synthesized to formulate MRF, and its field-dependent properties such as reversible transition were investigated [33]. Notably, the effect of the Fe_3_O_4_ on sedimentation stability was evaluated and compared with CIP-based fluid. It has been identified from this work that the fluid consisting of Fe_3_O_4_ can provide both high MR effects such as a higher storage modulus and better sedimentation stability compared with the CIP-based fluid. The better sedimentation can be primarily attributed to the decreased density mismatch between the proposed nanospheres and silicone oil. However, the wear characteristics and durability of the particles need to be tested further. Yang et al. [34] investigated the sedimentation of an MRF with micro-sized magnetic particles dispersed in oil carriers. Oleic acid and dimer acid were employed to adjust the hydrophobicity of the iron particles. The polar attractions between the dimer acid-covered particles caused a considerable increase in viscosity, indicating that a flocculation structure developed in the suspensions. Both the quick recovery of the viscosity and the higher viscosity–temperature index suggest the existence of particle–particle polar interaction in the suspensions containing dimer acid. It has been identified that the steric repulsion of oleic acid plays a limited role in the stability of suspensions only if a large quantity of surfactant is used. This result indicates that MRF with the dimer acid-covered particles have better sedimentation stability with the proper surfactants. Three samples were made to investigate the dispersibility effect of magnetic nanoparticles as a carrier, keeping the field-dependent MR effect as high as possible [35]. The samples were classified as follows: MR #1 contains CIP particles containing aluminum stearate (Aldrich), MR #2 contains CIP and a viscosity modifier to improve its stability, and MR #3 contains nanoparticles (ferrofluid) used as a carrier with the nanoparticles having the average size of 7.8 ± 0.3 nm. It was found that the field-dependent yield stresses of the three samples are almost the same, but the sedimentation properties are different. It was found after 30 days that MR #3 shows minimum penetration force, and it was confirmed that the halo of the nanoparticles keeps the iron microspheres away from each other, which can prevent irreversible aggregation by either van der Waals or magnetic attraction. There are many other methods used to reduce the impact of the sedimentation problem by treating magnetic particles. The recipes or methods presented and discussed in this review article are frequently used and cited by experts working on the development of advanced MRFs that show high sedimentation stability as well as prominent MR effects. Bossis et al. [36] investigated a fundamental network of particles that was the basic phenomenon responsible for the strength of the solid phase. In particular, this paper focused on the interplay between magnetic forces that are responsible for the gelling of the suspension as well as on the hydrodynamic and thermal forces that contribute to breaking this gel and allow the suspension to flow. The combination of these three forces gives rise to a very rich rheology whose many aspects are still not understood. Therefore, the main phenomena of several MRFs, the field-dependent rheological properties, and mathematical models in relation to the yield stress and the shear rate were analyzed. Lopez-Lopez and Vicente [37] investigated the sedimentation behavior of an MRF consisting of two extremely different particle sizes. Micron-sized magnetic particles were employed. To undertake this, they made a sample using a non-polar ferrofluid as a carrier fluid and micron-sized iron particles or magnetite nanoparticles. It was found that chainlike structures are not observed in the absence of an external magnetic field since the steric repulsion that imparts the oleate ions is adsorbed on the magnetite nanoparticles. It was also observed that the sedimentation rate from the electromotive force was induced in a sensing coil positioned around the settling suspension. The test result shows that the use of extremely bimodal suspensions, composed of micron-sized iron and nano-sized particles, exhibits decelerated settling and improves the redispersibility of magnetorheological fluids. Table 1 presents several recipes to enhance sedimentation stability by modifying magnetic particles. 

### 2.2. Modification of Carrier Liquids

**Figure 3:**
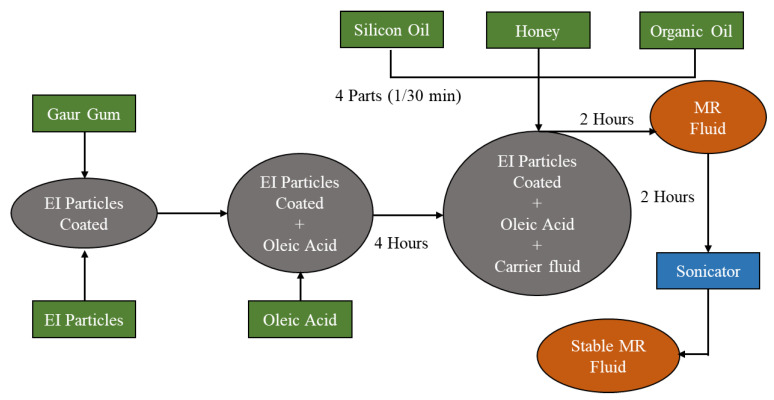
The manufacturing process of magnetorheological fluids featuring various base oils [39].

There are many possible carrier liquids that can be mixed with magnetic particles to formulate MRFs. The most critical problem during mixing is the inevitable particle sedimentation due to the density mismatch between the carrier liquid and particles. Therefore, many studies have been carried out to modify the carrier liquid to reduce sedimentation. In the early stages, many researchers tried to decrease the viscosity and increase the density of the carrier liquid to reduce the density mismatch. The studies on sedimentation stability include the use of combination oils, the optimization of the oil viscosity and particle volume fraction, and the use of green (or natural) carrier liquids such as Mahua oil. It is remarked here that wettability always exists between mutually incompatible liquids and solids, which is called the wettability or degree of wetting, which indicates the balance of interfacial forces between two materials (one liquid and one solid, most likely). Thus, the wettability of carbonyl iron powder and carrier liquid needs to be carefully treated as an important reference for the study of anti-sedimentation stability. R. R. Vannarth et al. [38] made MRF samples using natural oils as a carrier liquid instead of conventional silicone oil to investigate sedimentation stability. Two natural oils, Mahua oil and Simarouba oil, which are edible, were mixed with CI particles and electrolytic iron to make so-called green fluid. After preparing several samples by changing the particle sizes and carrier liquid, the sedimentation was tested as a function of time (250 h). It was evaluated when the fluid was allowed to settle only under the action of gravity. The particles in the fluid using Mahua oil as the carrier fluid settle quickly as compared to those in the fluids using Simarouba oil as the carrier fluid and silicone-based fluid. It was also observed that the sedimentation with the Simarouba carrier liquid is better than the others since the density of Simarouba oil (966 Kg m^−3^) is greater than that of the silicone oil (959 Kg m^−3^) and Mahua oil (956 Kg m^−3^). It is known that most MRFs are made using silicone oil as a carrier liquid since it is chemically stable and its MR effect is high. However, its sedimentation stability is not sufficient for practical implementation; hence, several carrier liquids need to be developed. In [39], two different natural oils, sunflower oil and cottonseed oil, and electrolytic iron powder coated with guar gum are used to make MRF. Figure 3 presents the manufacturing process of the fluids with various oils such as sunflower oil and cottonseed oil. In fact, the particles are coated by guar gum and mixed with oleic acid to achieve proper bonding on the surface of electrolytic iron (EI). It has been identified that the cottonseed oil blend-based fluid shows about 10% improvement over the sedimentation rate of the silicone oil-based fluid. Harsh et al. [40] used three different base oils that were e mixed with CIPs (the average size is 3 microns) to make the fluid. The three base oils include silicone oil (57.5%), lubricant oil (73%), and grease (78.5%). It has been identified that the three samples exhibit no sedimentation after immediately mixing, but the sample using the silicone oil shows the least sedimentation after being left out for 15 h. Another interesting observation is that the samples mixed with lubricant and grease start to sediment within two and four hours, respectively. However, the sample with the silicone oil starts to sediment after 24 h. Despite these results, the sedimentation rate of the three samples is too high for practical devices and systems. The high sedimentation stability of MRFs can reduce the agglomeration of ferromagnetic particles, prevent the irreversibility of the process, and keep them in an evenly dispersed status. It has already been reported that these inherent characteristics depend on several factors, including the properties of carrier liquids. S. Zhibin et al. [41] adopted five different types of lubricants to investigate sedimentation stability: hydrogenated castor oil, Teflon, boron nitride, molybdenum disulfide, and graphite. Firstly, the zero-field viscosity of the samples was tested by a viscometer, and the strength of viscosity under the zero-field condition was tested and compared. The sedimentation has been tested by measuring the size of the contact angle, which indicates the anti-sedimentation stability of the samples under different lubricants. Among the five lubricants, molybdenum disulfide has the smallest contact angle, the best wetting effect, and the highest zero-field viscosity. Therefore, it was found that molybdenum disulfide as a lubricant can effectively improve the anti-sedimentation stability of the fluids. Thus, the general rules of the addition of the lubricants used in this study can be adopted to observe sedimentation stability. Table 2 presents both typical base oils and newly developed recipes for carrier liquids that enhance sedimentation stability. 

### 2.3. Use of Additives

**Figure 4:**
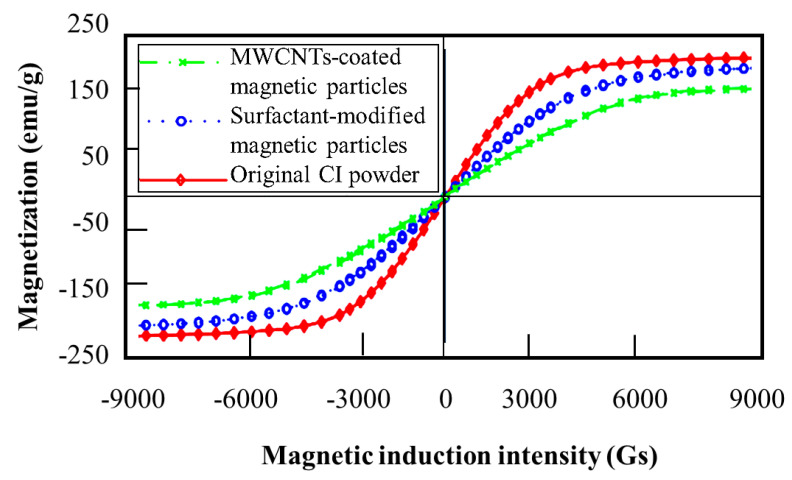
Comparison of magnetization curves of three magnetic particles (1 Gs = 0.0795775 kA = m) [49].

Besides the modification of the particles and base oils to reduce sedimentation, there are many studies on the use of additives to improve sedimentation stability. Lopez-Lopez et al. [42] investigated the aggregation, sedimentation, and re-dispersibility of concentrated iron-based MRFs using three different additives: oleic acid, aluminum stearate, and silica nanoparticles. It has been observed from the sedimentation test that although the addition of oleic acid or aluminum stearate cannot reduce particle settling, the re-dispersibility of the suspensions is considerably enhanced. It has also been found that the silica nanoparticles behave as a gel-forming agent capable of preventing particles settling under rest conditions. However, this causes the formation of compact sediments, making re-dispersion extremely difficult. The sedimentation behaviour of MRFs with polymer additives was studied in [43]. In this work, after observing the MR effect of the suspensions stabilized by two different polymers in terms of the pH number and magnetic field intensity, the sedimentation was tested. It has been identified that the proper use of the polymer additives can increase the stability under the magnetic field. This is reasonable since the hydrophobic/hydrophilic balance of the polymer affects the magnetic field’s ability to form magnetic structures by the aggregation of the magnetized particles. On the other hand, the field-dependent yield stress may be reduced by adding the additives because the presence of the stabilizing polyelectrolyte may deteriorate the magnetic field’s effectiveness when the relative proportion of both the particles and polymer confers optimum stability to the dispersions. Therefore, the trade-off between the sedimentation improvement and MR effect reduction should be carefully considered by considering the number and type of additives. Lita et al. [44] used three different commercial additives to improve the sedimentation rate of MRF via the dispersion and stabilization of iron particles, which were mixed with the synthetic oil carrier. Samples of the proposed substance called MRF-LM5 were prepared, which consists of a micro-powder of iron (99.5% Fe, 80 g) with particle sizes between 4–6 mm, and three different carrier liquids (20 g) of Castrol oil, transformer oil, and silicon oil. In addition, three different additives of denoted Thix (1.5 g), HT (1 g), and Pur 8050 (3 g) were added. It was found that the sedimentation rate of MRF-LM5 is twofold slower than that of a commercial sample of MRF-132DG made by Lord Corporation under the same magnetic field intensity. This result directly indicates that an appropriate use of the additives adaptable with magnetic particles and base oils can enhance sedimentation stability. Zhang et al. [45] prepared eight different MRF samples utilizing different sizes of the magnetic particles, different carrier liquids, and seven different additives to investigate sedimentation stability as well as the field-dependent properties such as the yield stress. The seven additives used in this work are (i) Tween-60, (ii) Span-60, (iii) Tween-80, (iv) Soan-80, (v) OP, (vi) oleic acid, and (vii) stearic acid. From the sedimentation test, it has been observed that the sedimentation stability of the sample with additives of Tween-80 and Span-80 is better than that of the two other samples with additives of OP and oleic acid, or Tween-60 and Span-60. In addition, it was observed that the sedimentary stability of the samples with a particle diameter of 2.73 μm is the best among the three kinds of diameters. Stearic acid can also increase the viscosity and sedimentary stability of the fluid, but the field-dependent yield stress is not improved. A study on the relationship between sedimentation and low-molecular weight has been carried out in [46]. The authors prepared a series of samples of magnetic fluids that were stabilized with low-molecular weight polypropylene glycol (PPG) of different molecular masses. The use of PPG can enable the maximum extension of the carrier fluid range to include ethyl- and butyl-acetate, ethanol, butanol, acetone, carbon tetrachloride, toluene, kerosene, and PPG itself. It has been observed at critical temperatures of other base fluids that alcohols are the best carrier medium. On the other hand, the coagulation stability of the ethanol-based ferro colloid with respect to water and kerosene was tested and it was found that the PPG-stabilized fluid partially coagulates in the presence of a small amount of water, after which the coarse particles dominate the solution. This effect can be applied to separate the magnetic colloid into fractions, thereby improving the sedimentation stability of the fluids. The additives are also used to improve the dispersion stability of MRFs. Fang et al. [47] used a dense network of multi-walled carbon nanotubes (MWCNTs) on the surface of CI particles to improve dispersion. In this synthesis, 4-aminobenzoic acid was used as a grafting agent through a two-step method: ultrasonication and solvent casting. The suspension stability was tested using the Turbiscan method, and it was identified that the field-dependent yield stress of the proposed sample is less than pure CIP-based MRF, but the sedimentation stability of the proposed sample is much better than the pure CIP-based fluid via reducing the density along with the rough surface of the particles. Both silane-coupling agent and bentonite additives have been used to enhance sedimentation stability via a new preparation process that is optimized through an orthogonal experimental design. The sedimentation ratio, apparent viscosity, and yield stress were measured to evaluate the performance of the sample prepared by the optimized process [48]. It was demonstrated that the proposed sample exhibited enhanced sedimentation stability when the silane-coupling agent and bentonite mass fractions are 2.88% and 3.60%, respectively. Moreover, the effect of the second stirring process on sedimentation stability was found to be more important than the first. The optimal preparation parameters to prepare the high-performance fluid were determined as follows: the first stirring time is 5 min and the second stirring time is 5 h at the stirring temperature of 20 °C, while both the first and second stirring procedures’ rotation speed is 2000 rpm. It has also been reported that the use of coated particles with additives are frequently adopted to improve sedimentation stability. Xu et al. [49] investigated the inherent properties of some samples including the yield stress and sedimentation using two kinds of coated magnetic particles: multiwalled carbon nanotube- (MWCNTs) coated magnetic particles and surfactant-modified magnetic particles. Then, these samples were classified into a group based on mixing the coated magnetic particles with different mixing ratios of coated magnetic particles, different volumes of the fractions, and different dosages of a thixotropic agent. It was identified from the test that the density of the two coated particles decreased. More specifically, the densities of the MWCNTs-coated magnetic particles and the surfactant-modified magnetic particles were decreased by 63.1 and 43.8%, respectively. The sedimentation stability of the samples with coated particles was increased. Figure 4 presents the magnetization curve of three different particles: MWCNT-coated particles, surfactant-modified particles (coated), and original CI particles [49]. It was found that the saturation magnetization of the original CI particles is 217 electromagnetic units per gram (emu = g), but the saturation magnetization values of the MWCNT-coated magnetic particles and surfactant-modified magnetic particles were 170 and 201 (emu=g), respectively. The coating materials are nonmagnetic, resulting in a decrease in the magnetization of the coated magnetic particles, although they still have good magnetic properties compared with other magnetic particles such as hematite, magnetite, and maghemite. Therefore, the prepared coated magnetic particles are very appropriate for the preparation of MR fluids. This magnetization property of coated particles results in the improvement of sedimentation stability. In order to investigate the influence of both additives on the rheological and sedimentary properties of the samples, stearic acid, sodium dodecyl sulfate (SDS), and their mixture were chosen for analysis [50]. The MRF was then composed utilizing CIP, silicone oil, liquid paraffin, graphite particles, bentonite, stearic acid, and SDS. In the first test, the field-dependent shear stress was observed, wherein the shear stress of the sample with stearic acid was greater than that of the sample with SDS, and the maximum increment was 73.81% when the mass fractions of CIP and additive were the same. However, when the mass fraction of carbonyl iron particles was 40~50%, the shear stress of the sample increased firstly and then decreased with the increase in the external magnetic flux density. On the other hand, it has also been found that when the mass fraction of carbonyl iron particles is 60~70%, the shear stress of the sample increased firstly and then became stable with the increase in the external magnetic flux density. The results directly indicate that the sedimentation stability of the sample with the mixture is better than that of the sample with the stearic acid and SDS. Specifically, the settling rate of the sample with the mixture increased by 91.53% compared to the other additives. Clay-based additives are also used for improving sedimentation stability because the dependency of the clay-based additive concentration on the sedimentation stability and the rheological properties of MRFs in non-activated state can be easily predicted [51]. Several samples were prepared for sedimentation testing: two different base oil viscosities, two different carbonyl iron particle sizes, and additive concentrations from 2 to 6 wt%. It has been identified from the experiment that the sedimentation rate decreases with the additive concentration, while the yield stress increases. The measurements of the rheological properties also showed the dependency of the rheological properties of the samples with a clay-based additive on the loading history. The influence of the carrier fluid viscosity or particle size has a minor effect on the sedimentation in comparison with the clay-based additive. The addition of 6 wt% additive slows down the sedimentation by more than 3000 times compared to an MR fluid without additives. In addition, the sample with 4.85% of clay-based additive exhibits slightly better sedimentation stability than the commercial fluid LORD MRF122. Li et al. [52] proposed a new method to characterize the sedimentation stability of MRF based on the change in the shear yield stress during the sedimentation process of the fluid. They prepared samples using three different surfactants containing dodecyl benzoate, polyethylene glycol, and oleic acid. From the test, it was found that the results show that oleic acid has the best effect on improving sedimentation stability. The change law of the shear yield stress of the sample in the next 90 days could be successfully predicted by fitting experimental data based on the least squares method. More exactly, the error between the test value and the fitting value is within 3% after 60–75 days, showing the reliability of the proposed surfactants to improve sedimentation stability. Regarding the addition of the additives to MRF to enhance sedimentation stability, the occurrence of the oxidation of particles may worsen the environment when MRF is practically implemented in many application systems. Therefore, Narwade et al. [53] proposed the use of so-called green additives to improve the sedimentation and agglomeration of particles of MRF, namely, guar gum and xanthan gum. After reviewing the methodology to prepare the fluid along with the process for adding the selected additives, it was found from the test that an improvement in the reduction in sedimentation was shown owing to the incorporation of the additives. In addition, it has also been observed that the sedimentation ratio depends on the additives, the carrier fluid, and the density of the carrier fluid and iron particles. The density of the iron particles should be as low as possible. The addition of thixotropic materials such as guar gum coating increases the yield stress of fluid and reduces the sedimentation ratio. Furthermore, it was found that the sedimentation stability can be enhanced by using fumed silica (Aerosil 200) and cottonseed oil as a carrier fluid. The optimization of the ratio of the particles to the additives needs to be further investigated to achieve the best sedimentation stability. Table 3 summarizes the types of the additives and recipes regarding the reduction in the particles’ sedimentation.

### 2.4. Use of Combined Recipes

As reviewed and discussed, one specific recipe may be sufficient for improving the sedimentation stability. However, a trade-off between the MR effect and sedimentation stability has occurred in many recipes proposed so far. Therefore, recently, combined (or hybrid) strategies consisting of more than two recipes have been studied [54]. In this section, some combined recipes are surveyed and discussed, focusing on the sedimentation of magnetizable particles. Among many factors, the most important aspect of enhancing sedimentation stability is to decrease the density of the magnetizable particles by coating them, increasing the viscosity of the carrier fluid using high-viscose liquids, using nanostructure materials, and modifying particles’ surfaces by adding a stabilizer surfactant. Notably, the coating of the magnetizable particles is one possible method to reduce interactions between the particles as well as particle density. Thus, by using this method, stability can be enhanced considerably, although the MR effect will normally be reduced. Recently, coated carbonyl iron particles with polymeric materials have been used more frequently. Another stabilization method is to use two different sizes of magnetic particles, namely, a combination of nanoparticles and microparticles in MRFs, in which the existence of nanoparticles in the base fluid increases the viscosity of the base fluid and thus reduces sedimentation. Some research studies focused on the optimal choice of the additives for improving both sedimentation stability and the MR effect. So far, several additives and surfactants such as stearic acid, organoclay, and fumed silica are used for suitable materials for increasing the sedimentation stability. One more interesting strategy for improving MRs’ effect and sedimentation stability is using a combination of ferrofluids and magnetorheological fluids associated with nanowires and the addition of proper stabilizers. With respect to this aspect, several studies have been reported. As for the ferrofluid-based magnetorheological fluid, Yang et al. [55] investigated the field-dependent rheological properties and sedimentation behavior. It has been determined that the static yield stress is increased as the weight fraction increases and the sedimentation stability is increased with the addition of a ferrofluid, which has anti-sedimentation properties. Susan-Resiga and Vekas [56] also studied the rheological properties of ferrofluid-based magnetorheological fluids. They focused on the analysis of the Mason number (Mn) defined for these nano-micro composite fluids and the corresponding Casson (Ca) number. From the analysis, it was found that the ferrofluid-based nano-micro composite fluids exhibit improved field-controlled responses, kinetic stability, and redispersibility, including when applied to very-high-magnetization-sealing fluids. It has also been demonstrated that the proposed sample can offer agglomerate-free, high-colloidal stability, maintaining Newtonian behavior in the absence of a magnetic field and a high MR effect under on-state conditions. By means of controlling the particle concentration at both hierarchical levels, nano and micro, the magnetic MR properties can be tailored to offer a high-performance lubricant for application systems such as dampers, brakes, and rotating seals. Rosensweig [57] derived expressions for small particle cloud formation leading to large particle repulsion in magnetorheological suspensions in a ferrofluid. In general, a suspension of relatively large micron-size particles dispersed in a ferrofluid of much smaller particles yields a magnetorheological fluid with superior properties. In the absence of an applied field, the experiment reveals the presence of a nanoparticle cloud surrounding each micron-sized particle. It has been identified from this work that Van der Waals forces predominate over magnetic dipolar forces in forming the clouds. In addition, a further analysis revealed that a repulsion force arises due to the squeezing of the clouds as two cloud-surrounded particles approach one another. Repulsion aids the redispersion of the larger particles by helping to prevent their agglomeration. The general recipes for the improvement of sedimentation stability entail the use of a surfactant and stabilizer, an organic polymer wrapped in the surface of the magnetic particles, coated soft magnetic particles, a magnetic particle surface coated with a layer of metal powder, adjusting the ball feed ratio and milling time appropriately, and adding nanomaterials [58]. It was demonstrated that the sedimentation stability is enhanced by adding appropriate additives and surfactants, adding magnetic particles coated with an organic polymer, adding composite magnetic particles, incorporating some nanomaterials, adding some oxidants, and so forth. Many types of MRFs are made using three components, including a base fluid, metal particles, additives, and lithium grease to reduce sedimentation. In one study, sedimentation properties were studied by visual inspection [59]. It was demonstrated that that the percentage of grease affects the sedimentation ratio. The higher the content of grease, the lower the degree of sedimentation, and vice versa. This directly indicates that the percentage of grease affects sedimentation behaviour. In addition, the effects of temperature on the viscosity of the prepared fluid samples were investigated. The viscosity of the sample plays an important role in its function at time-varying temperatures. The greater the iron content, the higher the viscosity. On the other hand, at different temperatures, the value of viscosity has been calculated and the effects of temperature on viscosity have been observed. Morillas and Vicente [60] comprehensively reviewed the magnetic materials including MR fluid, MR elastomer, and foamed MR elastomer in terms of the preparation recipe, field-dependent rheological properties, sedimentation stability, appropriate applications, and future challenging issues for practical implementation. Several methods to reduce particles’ sedimentation have been discussed. The first method is to mix the micro-sized particles with the nano-sized particles (large-to-small diameter ratio is around 1000); consequently, the MRF suffers from Brownian motion interacting with the main population and this hinders the sedimentation of the latter. However, it should be noted that in this case, the on-state yield stress is not improved for any volume fraction of the nanoparticle concentration. The second approach is to create a bimodal MRF with a large-to-small diameter ratio of 100, in which the minority population of the particles has a mean diameter around 100 nm, just in the transition between the magnetic single-and multi-domain regimes. Thus, both the magnetic interactions and yield stress are enhanced in the on-state and sedimentation rate becomes slower with better re-dispersibility in the off-state. Another interesting approach to reduce sedimentation is to use a dimorphic MR fluid that has different particle shapes. For example, dimorphic samples consisting of sphere- and rod-shaped particles show that rod particles expose a larger wetted area that makes them aggregate in order to reduce surface energy and experience more contact points with other particles. This behaviour increase the stress bearing structure that interlocks the spheres forming a secondary structure. Therefore, both the off-state yield stress and sedimentation stability are enhanced in the off-state. Sometimes we need an MRF that is usable at high temperatures. Li et al. [61] made such a sample utilizing soft magnetic particles, surfactants, and base carrier fluids. Then, its field-dependent rheological properties and sedimentation stability were evaluated. The magnetic particles with a high Curie temperature showed an excellent stability at high temperatures. It was found from the experimental test that the proposed sample manufactured for high temperatures exhibits high sedimentation stability. In particular, a long-time heating at 200 °C leads to a higher sedimentation rate. However, the negative performance can be offset by stirring due to shearing in application systems and devices. On the other hand, Thiagarajan et al. [62] presented a broad overview of the most recent breakthroughs in MRF chemical, colloidal, and thermal stability as well as an improvement of the sedimentation stability in MR performance through the engineering of new materials. In particular, the effect of the particles, additives, and coating materials on the sedimentation stability was discussed in detail. It was found that a coating of nonmagnetic materials on magnetic particles led to a decrease in sedimentation stability with a simultaneous decrease in the MR’s effect. The addition of certain organic or inorganic additives were found to increase both the yield stress and colloidal stability to an extent, but the detailed behaviour needs to be further investigated. It has been remarked that additional challenging work regarding the prevention or reduction in sedimentation problems during the operation of MRF-based applications need to be explored further in the future. Unlike the previous studies focusing on the passive method for improving the sedimentation stability of MRF, recently, Zou et al. [63] proposed an active dispersing mechanism (ADM), which is one of attractive ways to resolve the sedimentation problem in the level of a device and, hence, it is promising for achieving good serviceability for MR dampers, even if a small degree of settling remains. To accomplish this target, rotary blades were employed to achieve the redispersal of the settled MR fluid under the theory of solid–liquid two phase flow. The parameters and working conditions of the rotary blades were optimized to guide experimental verification in a damper-sized vessel. The vessel can be seen as a prototype for a real MR damper. An immersed induction method for the characterization of the localized fluid concentration was proposed to designate the dispersal process when the ADM is started. Through experiments that employed different fluid volume fractions and rotating speeds of the rotary blades, it was fully demonstrated that the faster the blades rotate and the shorter the mixing time, the better the dispersion capability. In addition, the rotary blades with optimized parameters can disperse the fully settled MR fluid with an initial volume concentration as high as 30%. This is possible by adjusting both the blade angle and rotational speed. Therefore, the ADM proposed in [63] is very effective for dispersing the settled MR fluid and promising with respect to the sedimentation immunity of application systems such as an MR damper. Table 4 summarizes the methods used to improve sedimentation stability using combined methods with more than two recipes.

## 3. Challenging Works

### 3.1. Sedimentation Test

**Figure 5:**
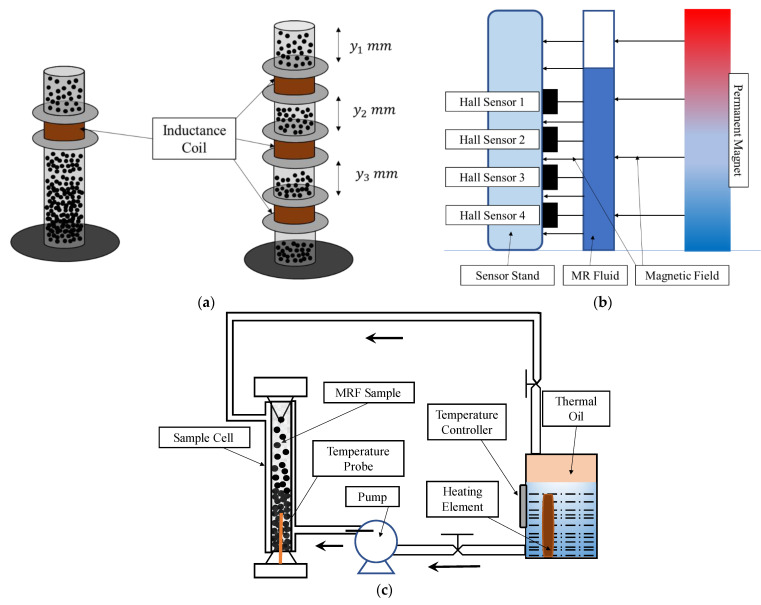
Measurement Methods for Sedimentation Rate of MRFs; (**a**) use of inductance coil, (**b**) use of hall sensor, and (**c**) use of thermal conductivity.

Regarding sedimentation tests, there are several methods, but there is no international standard method that stipulates the test conditions and the results’ evaluation. The sedimentation stability is traditionally first investigated by visual inspection via the naked eye. However, with visual observation, it is not possible to identify any internal stratification in the MRF because the fluid is stratified in four sedimentation zones: a supernatant zone, an original concentration zone, a variable concentration zone, and a sediment zone. Therefore, more sophisticated measurement methods are required, with which the sedimentation profile can be tracked as a function of time. During sedimentation, the settling of particles leads to the formation of two distinct regions in MRFs: a turbid region at the bottom (due to the presence of particles) and clear region at the top (due to the absence of particles). This is quantified through a “sedimentation ratio,” which is defined as the ratio of the height or volume of the dispersed/turbid phase to the total height or volume of the MRF and is often calculated over time. It is known that a higher sedimentation ratio indicates the reduced settling of particles and increased fluid stability. The sedimentation ratio is often determined by visual inspection, a hall sensor, ultra-violet (UV) spectroscopy, laser beam deflection, turbidity measurement, or thermal conductivity. Although the sedimentation ratio quantifies the colloidal stability of the fluid, as mentioned earlier, it must be noted that there is no standard method to determine the sedimentation ratio. The sedimentation ratio is calculated between different time intervals and using different techniques, and hence a careful comparison is required to evaluate its measurement accuracy. Iglesias et al. [64] presented an experimental test method for the sedimentation rate of MRF based on the measurement of the inductance of one or more sensing coils located at specified positions around a test tube containing the suspension, as shown in Figure 5a. Such a measurement method is more accurate than visual observation. A proper electrical circuit consisting of an inductor and capacitor can be used to easily measure the resonant frequency, and the relationship between the resonant frequency and the volume fraction of the particles is determined and set at the coil’s location. Thusly, the sedimentation kinetics of iron suspensions in base fluids of viscosities and volume fractions equivalent to solids are calibrated. Therefore, this test method can be used to accurately measure the volume fraction of solids in concentrated suspensions at one or more locations in the suspension container as a fraction of time. The ability of the device to detect the settling of iron suspensions with volume fractions up to 25% has been proposed in [65], in which hall sensors are used. To quantify the settling of the particles, an experimental setup used to measure the variation in magnetic strength caused by the change in the permeability of the settled particles was established, as shown in Figure 5b. In this experiment, a 1 T Neodymium magnet was used to generate a uniform magnetic field. When the MR particles start to settle, the permeability of the solution decreases at the top and increases at the bottom, and such a change in the magnetic strength was measured using four numbers of hall sensors located at different positions. Cheng et al. [66] proposed a sedimentation measurement method for characterizing the sedimentation rate in an MRF column utilizing thermal conductivity correlated with the particle concentration, as shown in Figure 5c. The results of this test can be summarized as follows: a series of samples composed of carbonyl iron particles suspended in silicone oil were prepared, and their concentrations (measured as volume fractions) and thermal conductivities were tested. A calibration curve was developed to relate the particle concentration to the thermal conductivity using this set of the samples with known concentrations. The particle concentration in the MRF column was then monitored by measuring the thermal conductivities at a fixed location and using this calibration relationship. Finally, the sedimentation rate in the MRF column was determined by examining how the particle concentration varied with time. The sedimentation rate measured in the column was validated using visual observation of the mudline (the boundary between the topmost clarified fluid zone and the MRF below). Recognizing that there is a simple linear relationship between the particle concentration and thermal conductivities in the MRF leads to a straightforward method of characterizing sedimentation in the column. Using this method, the particle concentrations of the MRF30 and MRF40 samples were successfully monitored over time at the sensor’s location. The sedimentation rate of both samples was calculated by computing the rate of change of the particle concentration as a function of time. To validate these results, the sedimentation rates for both fluids were compared to the results obtained using visual observation, and the relative error between these two methods was −1.408% for MRF30 and 2.04% for MRF40, respectively. Therefore, the measurement method proposed in this work establishes the feasibility of using thermal conductivity measurements to monitor both the particle concentration and sedimentation rate in the column. Choi et al. [67] introduced a new method for the measurement of the sedimentation of MRFs. They proposed a vertical measurement setup using a vertical axis inductance-monitoring system where they measured the settling velocity of MTF with respect to the particle concentration and compared it with other methods such as the Dick model. Later, Wen et al. [68] extended the previous work [63] to monitor the sedimentation using a vertical axis-monitoring system with a low-aspect ratio sensor coil. The experimental apparatus can measure the inductance signal from the inductance sensor and translate it up and down the vertical MRF column to track the mud-line, which is defined as the boundary between the clarified fluid at the top of the column and the MRF below. This method is more accurate than the previous method since the time history of the divided sedimentation zone can be measured. Notably, so far, the formulation of an international standard method for determining the sedimentation rate (or ratio) in MRFs has not been reported. This is a challenging issue since so many factors influencing the sedimentation need to be considered, namely, standard instruments, the number of particles, the base oil volume, the testing period, the temperature and moisture conditions, the dispersing properties, a calculation formula for sedimentation, and so forth. 

### 3.2. Sedimentation in Storage and Practical Use

Currently, several MRFs, including commercially available ones, are practically usable for certain applications such as MR dampers for vehicle suspension systems due to the sufficient damping force and response time. However, there is still a great deal of challenging work to be performed in order to improve the salient properties of MRFs, which are required for commercialization to make various semi-active control systems. Such work includes that related to sedimentation stability, particle oxidation for a long time, chemical stability, high-temperature instability, colloidal stability, low viscosity in the off-state, high yield stress in the on-state, toxicity, biodegradability, and environmental pollution concerning the amount of waste, low power consumption, low cost, and sealing durability. Among these problems, sedimentation stability is the one that most needs to be resolved for successful commercialization. So far, many different recipes or treatment methods to enhance sedimentation MRFs have been comprehensively reviewed and discussed in this review article. Therefore, the storage methods of bottles filled in MRFs and practical applications to reduce sedimentation are conceptually introduced in this section. To the author’s knowledge, there has not yet been a report on this issue with respect to the sedimentation problem of MRF-filled bottles (called bottles) and MRF-filled application devices and systems (called applications).

**Figure 6:**
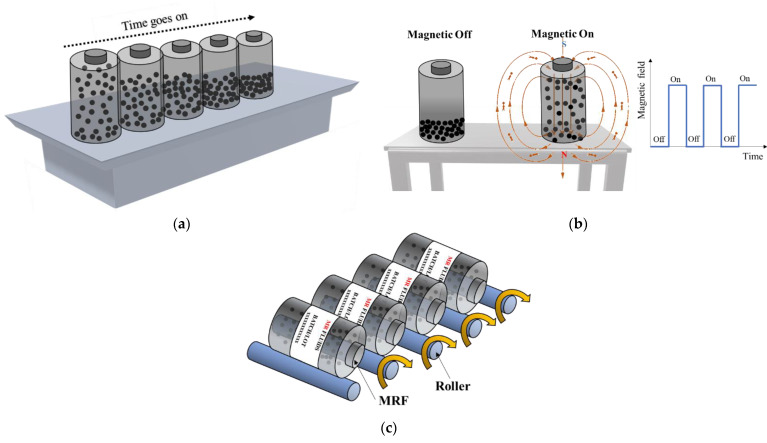
Conceptual methods for sedimentation reduction in bottle storage; (**a**) vertical standing on shelf, (**b**) vertical standing with on–off field state, and (**c**) horizontal position in rolling state.

**Figure 7:**
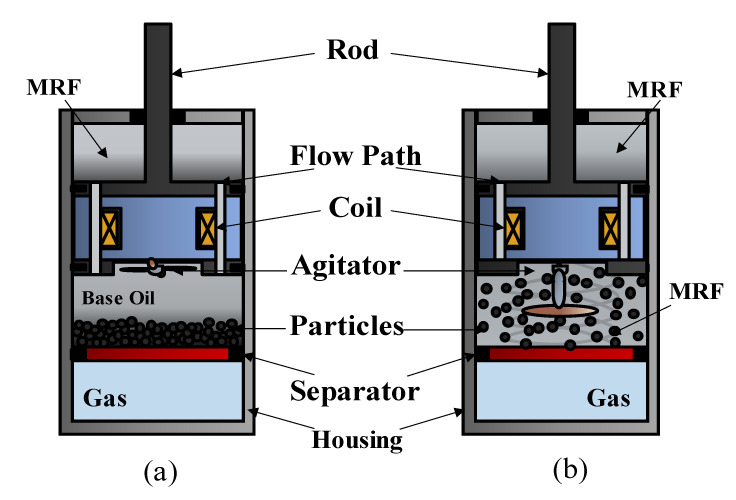
Conceptual method to disperse the settled particles in MR damper that has rested for a long time without operation; (**a**) no operation for a long time; (**b**) starting operation after long rest.

Figure 6 shows three methods to store the bottles: the storage of the bottles standing vertically on a shelf; the storage of the bottles with and without a magnetic field, in which an on–off switching controller is used; and the rolling of the bottles without the field. It is easily expected that the fastest particle sedimentation occurs in the vertically standing storage case. Notably, so far, there has not been a study on the sedimentation of MRFs depending upon a magnetic field. However, it is expected that the sedimentation behaviour of MRFs will change by applying magnetic intensity since the formation of the chain-like structure is changed every time depending on the field intensity and the on–off interval state. The storage of the bottles in a rolling machine will significantly reduce the particle sedimentation caused by the dynamic motion, but it is quite costly. Very recently, an active dispersing mechanism was introduced in [63]. The authors have proven that the settled particles are well-dispersed by the rotational blade attached to the inside of the cylinder. This method can be extended to applications to reduce particle sedimentation. Figure 7 presents a linear MR damper in which a tiny rotational blade is attached to the bottom of the piston. When the MR damper is being frequently operated, the blade is fixed. However, when the MR damper starts to operate after resting for a long time, the blades rotate at proper time intervals to stir the settled particles and start the operation. This can, of course, improve sedimentation stability, but the part of the piston where the rotating blades are attached should be modified, unlike a conventional MR damper. It should be noted that the development or creative design of many different types of MRFs or methods to enhance the sedimentation stability of MRFs from the bottles’ storage to practical applications constitute challenging studies, which consider many factors associated with the MRF itself as well as the operating conditions of application systems. It is evident from this review article that many comprehensive studies that analyse successful commercial products utilizing MRFs need to be carried out with respect to two approaches: one concerns the development of an advanced MRF exhibiting low sedimentation and a remarkable ER effect, and the other concerns preventing the sedimentation of MRF during its storage and application. The former method may be called a ‘passive method’, while the latter can be referred to as an ‘active method’ when the particle sedimentation of MRFs is studied. 

## 4. Conclusions

One of critical issues that must be resolved for practical applications of magnetorheological fluids is that of sedimentation. Therefore, in this review article, the causes of sedimentation were comprehensively reviewed in terms of the principal ingredients for making the fluids, and some recipes to enhance sedimentation stability were discussed and summarized. In addition, conceptual methodologies to prevent sedimentation from the bottles’ storage in application systems were suggested as future challenging research avenues for successful commercial products. In the particle modification procedure, the use of nanosized particles and the addition of spherical fillers with nanosized fumed silica have been identified as favorable recipes for improving sedimentation stability without degrading the MR effect. In the carrier liquid, the basic problem of the density mismatch between the particle and liquid was confirmed as the most serious problem to be resolved in terms of the sedimentation aspect. Therefore, several studies regarding density modification, the change of the kinematic viscosity, and the combination of silicone oil and oleic acid have been carried out. On the one hand, many studies on the use of appropriate additives or surfactants were undertaken to improve sedimentation stability while maintaining field-dependent rheological properties. Some of the additives such as oleic acid, aluminum stearate, acrylic acid polymers, silane-coupling agents, dimer acid, styrene fumarate copolymers, and clay-based additives have been found to be good candidates for mitigating the sedimentation problem. In addition, the use of coated particles with multiwall carbon nanotubes (MWCNT) has been evaluated as one of the solutions to resolving the sedimentation problem. Furthermore, the combined method using more than two recipes has been identified as one of the effective methods for reducing sedimentation. In the last section, some challenging future studies were briefly suggested and discussed. For example, to achieve data reliability of the sedimentation result, an international standard method for sedimentation measurement is required which considers the principal test conditions. So far, no such standard has been reported with respect to how to prevent or reduce the sedimentation of the fluids stored in bottles or filled in the application system. Therefore, in this review article, conceptual strategies to reduce the sedimentation from storage in application systems have been discussed, showing schematic diagrams. 

Finally, most of the scholars who are working on magnetorheological fluid technology in the fields of science, engineering, and technology surely agree that the most significant problem to be resolved for a successful commercial product is particle sedimentation. In this regard, this review paper will be very helpful to potential readers working in a related field for the creation of novel ideas to develop more advanced MRFs and commercial products.

## Figures and Tables

**Table 1:** Particle Modification for Improvement of Sedimentation Stability.

Typical Particles	Recipes
Iron Iron Oxide Iron Nitride Iron Carbide Carbonyl Iron Chromium dioxide Low Carbon Steel Silicon Steel Nickel Cobalt	(Fe_3_O_4_ with 5microns and 5.18 g/c3 density) + (small amount of nanosized ferromagnetic particles: Co-γ-Fe_2_O_3_ and CrO_2_) [20] (CIP with 4.5–5.2 microns and 4.4 g/cm^3^ density) + (spherical filler with the nanosized of 10 nm: fumed silica) [21] (CIP with higher 97% iron particles) → (treated with 0.5 M HCl for 1 h) → → (powder dried from residues for 6 h under a vacuum) → (bulk of the 100 g of surface-activated particles mixed with 3-neck flask with 250 mL of toluene) → (CI particles with amino groups on the surface) [22] (Micro-sized iron particles having a plate-like shape with density of 7.8 g/cm^3^ and size of 2–19 microns) → (control of particle volume fraction by mechanical stirrer) → (16 wt% volume bi-disperse MRF) → (observation of the disappearance of the aggregates) [23] (CIP particles) → (make two different size that are micron- (2 microns) and nano-sized (50 nm) → (nanoparticles are surface stabilized against oxidation with FeO–Fe_3_O_4_ double shell) → (2 wt% of lecithin is added and particles are composed of silicone oil and nanoparticles) [25] (MgFe_2_O_4_ nanocrystal clusters were synthesized with a surfactant-assisted solvothermal method) → (1.35 g FeCl_36_H_2_O and 0.51 g MgCl_2_$6H_2_O were dissolved in 50 mL ethylene glycol) → (1.00 g ascorbic acid and 3.20 g NaOH were added slowly) → (sealed tightly and washed with absolute ethanol and deionized water) → (The resulting magnetic materials were then dried in vacuum for 24 h) [26] (CI particles + Oleic acid (OA) + anhydrous alcohol stirred in a beaker for 1 h) → (washed with deionized water until the pH value of 7) → (CI particles with OA group were obtained: the volume fraction of the modified CI is from 10–28%) [31] (Hard CIP of 4.5–6 microns and density 7.8 g/cm^3^) → (thermal decomposition of iron pentacarbonyl [Fe (CO)_5_]) → (high content of other elements) → (annealing of HARD-grade carbonyl iron powder under hydrogen) → (the content of C, N, and O is reduced) [33] (HS-Fe_3_O_4_ particles were fabricated) → (encapsulation of pores among Fe_3_O_4_ primary nanoparticles resulted in a lower density of HS-Fe_3_O_4_ (ρ = 3.32 g/cm^3^) → (HS-Fe_3_O_4_ exhibits a greater saturation magnetization value) → (longer-term stability than MR fluids of bare Fe_3_O_4_ particles) [34] (Hierarchically structured mesoporous Fe_3_O_4_ nanospheres were synthesized) → magnetic properties of the as-synthesized Fe_3_O_4_ nanospheres were examined) → Fe_3_O_4_ nanospheres-based MR fluid demonstrated enhanced sedimentation stability: the finding was a particular mesoporous structure) [35]

**Table 2:** Base Oil Modification for Improvement of Sedimentation Stability.

Typical Base Oils	Recipes
Silicone Oil Polyalphaolefin (PAO) 1-ethyl-3-methylimidazolium Diethylphosphate 1-hexyl-3-methylimidazolium Chloride Hydrocarbon Oil	(1) Use of green base oils developed from trees [38]: Mahua oil (density at 30 °C: 956 kg/m^3^, kinematic viscosity at 40 °C: 52 Mm^2^/s) Simarouba oil (density at 30 °C: 966 kg/m^3^, kinematic viscosity at 40 °C: 56 Mm^2^/s) The density of simarouba oil (966 Kg m^−3^) is greater than that of the silicone oil (959 Kg m^−3^) and mahua oil (956 Kg m^−3^). (2) Use of eco-friendly base oil with low off-state viscosity [39]: Cottonseed oil (viscosity at 25 °C: 0.353 Pa.s, density at 25 °C: 926 kg/m^3^, and flash point: 315 °C) Sunflower oil (viscosity at 25 °C: 0.0353 Pa.s, density at 25 °C: 924 kg/m^3^, flash point: 315 °C) Honey: (viscosity at 25 °C: 9.3 Pa.s, density at 25 °C: 1386 kg/m^3^, and flash point: 40 °C) The rate of sedimentation of the sunflower blend-based MR fluid is faster than cottonseed blend-based MR fluid. (3) Effect of combined carrier liquids [40]: Use of the lubricant oil (73%) + Oleic acid 2% Use of grease (78.5%) + Oleic acid 1.5% Use of silicone oil (57.5%) + Oleic acid 2.5% The silicone oil with 40% of CIP and surfactant concentration of 2.5% by wt. shows the lowest sedimentation rate among the three samples. (4) Use of the lubricants [41]: Molybdenum disulfide (zero field viscosity: 955 mPa.s) Hydrogenated castor oil (zero field viscosity: 953 mPa.s) Boron nitride (zero field viscosity: 712 mPa.s) Graphite (zero field viscosity: 756 mPa.s)

**Table 3:** Additives for Improvement of Sedimentation Stability.

Typical Additives	Recipes
Thixotropic Agent Carboxylate Soap Antioxidant Lubricant Viscosity Modifier Metal-Oxide Powders Sulfur-Containing Thioesters Xanthan Gum Stearate—Carboxylic	(1) Effect of additives on sedimentation [42,43]: Oleic acid: degrades particle sedimentation and improves irreversible aggregation Aluminum stearate: increases the sedimentation rate with higher concentration Silica nanoparticles: the sedimentation rate decreases as the silica concentration increases Redispersibility can be improved by adding oleic acid and aluminum stearate Polymers: polymer concentrations have a very significant effect on stability Clay additive: the addition of 6 wt% improves sedimentation more than 3000 times compared with MR fluid without the additive. (2) Use of commercial additives [45]: Types of additives: Tween-60 and Span-60, OP and oleic acid, Tween-80 and Span-80, tween-80, span-80, Stearic acid Result: sedimentary stability of MR fluid with additives of Tween-80 and Span-80 is better than that of two other MR fluids with additives of OP and oleic acid, or Tween-60 and Span-60. (3) Effect of surfactants [48]: Surfactant: silane-coupling agent KH-550 (density of 0.94 g = mL, boiling point of 217 °C, and flash point of 150 °C.) Bentonite is used as a thixotropic, which has a relative density of 1.7 and melting point of 1100 °C with stable chemical properties. Optimization: 5.0 g surfactant (silane coupling agent KH-550), 4.0 g thixotropic agent (Bentonite), 30.0 g carrier fluid (silicone oil), and 100.0 g particles (CIP). Result: This prevented particle agglomeration and improved the sedimentation stability (4) Effect of coated particles [49]: MWCNTs-coated magnetic particles: Tap-density 1.55 g/cm^3^, reduction proportion: 63.1 % Surfactant-modified magnetic particles: Tap-density 2.36 g/cm^3^, reduction proportion: 43.8 % Result: The sedimentation rates with an optimal mixing ratio of coated MWCNTs can effectively reduce the sedimentation of MRFs (5) Three different MRF with additive mixtures [51]: MR #1: 0.4% aluminum stearate (AlSt) MR #2: 0.4% AlSt (the viscosity modifier is a mixture of two polymers: polyester and styrene-di(alkyl) fumarate block copolymer MR#3: 6.2% *v*/*v* volume fraction nanoparticles as a carrier fluid (ferrofluid) without other additives Result: MRF #3 shows minimum penetration force preventing their irreversible aggregation by either Van der Waals forces or magnetic attraction. (6) Use of three additives of stearic acid, sodium dodecyl sulfate (SDS), and their mixture [53]: Sample 1: CI 40% + SDS 2.0% +Silicone oil 54.0% Sample 2: CI 50% + SDS 3.0% + Silicone Oil 43% Sample 3: CI 60% + SDS 4.0% + Silicone Oil 32% Sample 4: CI 70% + SDS 5%+ Silicone oil 21% Results: The stability of MRF with the mixture increasingly improved with the increase in mass fraction of CI particles. However, when the mass fraction of CI particles was 50%, the stability of MRF decreased when the additives were stearic acid and SDS.

**Table 4:** Combined Methods to Improve the Sedimentation Stability.

References	Recipes
M. Ashtiani et al., 2014 [54]	Ball-milled electrolytic iron particles (1–5 microns) + poly alfa olefin oil+ coating with guar gum + oleic acid additive CIP (1-6 microns) + silicone oil + coating with guar gum + stearic acid additive Iron nano particles (30–50 nano-sized) particles + synthetic oil + coating with polyvinyl pyrrolidone + honey additive
F. Zhou et al., 2015 [58]	CIP (2-4 micro-sized + mineral oil + coating with Poly Methyl Methacrylate (PMMA) + submicron organoclays additive Iron oxide particles (7–8 micro-sized + silicone oil + coated with the polymer composite + aluminum stearate
H. Singh et al., 2016 [59]	Magnetic particles (CIP size adjustment) + (polymer-coated magnetic particles) + (ball-milled organic polymer) + base oils (silicone oil, mineral oil, sunflower oil, synthetic oil, cotton seed oil) + additives (sodium dodecyl benzene sulfonate, polyethylene glycol, oleic acid, carboxylic acid organic salt, alkylamine phosphate ester, silane coupling agent)
J.R. Morillas and J. Vicente, 2020 [60]	Modified particles (high-saturation magnetization of metal particles, iron particles of size less than 53μ, dispersed particles without the clustering) + modified base oil (magnetically neutral, temperature independent, neutral lubricating features) + modified additives (grease, lithium grease, stabilized surfactants): proper combination of three constituents
S. Thiagarajan and A. S. Koh, 2021 [62]	Improved particles (Iron oxide, Lithium zinc ferrite, manganese zinc ferrite, nickel zinc ferrite, cobalt nanofibers) + improved carrier liquids (silicone oil, sunflower, blend of silicon oil, honey, and cottonseed oil, PDMS) + additives (iron oxide, carbon globules synthesized from toluene, MWCNTs, graphene, copper, aluminum) + Coatings (octyl acyl ethylenediamine triacetate, stearyl acyl ethylenediamine triacetate)

## Data Availability

All data presented in this review article are available from the cited reference corresponding to each recipe to improve the sedimentation stability.
